# Systematic Client Feedback in Youth Mental Health and Addiction Care: A Controlled Study Comparing Two Treatment Cohorts

**DOI:** 10.1159/000528355

**Published:** 2023-01-17

**Authors:** Patty van Benthem, Renske Spijkerman, Peter Blanken, Albert Boon, Robert Vermeiren, Vincent Hendriks

**Affiliations:** ^a^Department of Child and Adolescent Psychiatry, LUMC Curium, Leiden University Medical Center, Leiden, The Netherlands; ^b^Parnassia Addiction Research Centre (PARC), Parnassia Academy, The Hague, The Netherlands; ^c^Youz, Parnassia Group, The Hague, The Netherlands

**Keywords:** Client feedback, Youth, Addiction care, Treatment outcome, Therapeutic alliance

## Abstract

**Introduction:**

We investigated the value of systematic client feedback in youth mental health and addiction care. In the present study, we examined whether a client feedback intervention would result in improved therapeutic alliance and treatment outcomes.

**Methods:**

Two hundred and four adolescents participated in the study using a − non-randomized − between-group A/B design. In the first study group, 127 patients were offered 4 months of treatment as usual (TAU), and in the second study group, 77 patients received the client feedback intervention as an add-on to TAU during 4 months.

**Results:**

Youths who received systematic client feedback in addition to TAU did not show better treatment outcomes or better alliance ratings after 4 months than youths receiving TAU only. Sensitivity analyses, in which we compared the more adherent patients of the second study group with patients receiving TAU, did not show significant beneficial effects of client feedback either. Also, the client feedback intervention did not result in lower rates of early treatment drop-out.

**Discussion/Conclusion:**

Our results cautiously suggest that client feedback does not have incremental effects on alliance and the treatment outcome for youth in mental health and addiction treatment. Moreover, our study highlights the challenges of implementing client feedback in clinical practice and the need for additional research addressing these challenges.

## Introduction

While psychological treatment can effectively reduce mental health and substance abuse problems in youth [1, 2, 3], a considerable proportion of youths does not (sufficiently) benefit from psychological interventions and/or leaves treatment prematurely [4, 5, 6]. During treatment, adolescents may show no progression, deteriorate, or drop out early without improvement in their primary complaints. Timely monitoring of treatment progress is thus needed to detect early insufficient symptom improvement or deterioration and to prevent early treatment drop-out by aligning treatment activities to the patient's needs. Monitoring of treatment usually focuses on symptom change but may also be directed at collecting and discussing client feedback about the therapeutic alliance [7]. To date, several studies have suggested that systematic monitoring of symptom change and of clients' judgments of the quality of the therapeutic alliance may have potential to improve the treatment outcome in mental health and addiction care [8, 9, 10, 11].

Systematic client feedback, in which the client regularly provides and discusses information pertaining to his/her well-being and perception of the quality of the therapeutic alliance with the therapist, can increase the effectiveness of the therapist and may encourage shared decision-making and a better treatment fit [12, 13]. The two most frequently used and studied client feedback questionnaires in clinical practice are the Outcome Rating Scale (ORS) and Session Rating Scale (SRS) [14, 15].

The effects of systematic client feedback on treatment outcomes in mental health care have been studied more often in adults than in youths and more often in mental health care than in addiction care. For adults, a Cochrane systematic review on routine outcome measurement and feedback in adult mental health care and pooled outcome data from 12 controlled studies including 3,696 patients showed no significant difference in the treatment outcome between feedback and non-feedback groups [16]. In a meta-analysis on the effectiveness of two frequently used client feedback systems, Lambert and colleagues [17] found small to moderate beneficial effects of feedback in adults with mental health problems that were treated in different settings, but these beneficial effects of feedback were not confirmed in a psychiatric setting [18]. In a recent review of 12 randomized controlled trials, no beneficial effect of feedback was found [19]. However, the most recent meta-analysis, which included 58 randomized and non-randomized studies, did find a − very small − effect of feedback on symptom reduction [20].

For youth, a Cochrane review on client feedback in psychological treatment for adolescents (11–18 years) with mental health problems included six controlled studies among 1,097 patients [21]. Due to high heterogeneity between studies and low comparability of assessments, data could not be pooled for meta-analysis. According to the authors, no firm conclusions could be drawn about the effects of client feedback for treatment outcomes in youth mental health care (YMHC) due to the lack of high-quality data and inconsistency of findings. None of the studies in this review involved youth in addiction care.

To summarize, client feedback has not yet received attention in youth addiction care (YAC), and based on the few studies on client feedback in YMHC, no firm conclusions can be drawn. In a previous study, we investigated the prognostic value of the therapeutic alliance for the treatment outcome in our study sample [11]. Our findings showed that the initial − first-session − therapeutic alliance was a strong predictor of treatment outcome: youths with a strong alliance according to both the youth and therapist perspectives had an eightfold odds of a favorable treatment outcome compared with youths with a weak alliance according to both perspectives. The aim of the present study was to further investigate the value of systematic client feedback for adolescents in youth mental health and addiction care. We examined whether treatment as usual (TAU) with the addition of a client feedback intervention in which youths provided and discussed information about his/her well-being − based on the ORS − and perception of the therapeutic relation − based on the SRS − with the therapist would result in a stronger therapeutic alliance and better treatment outcomes than TAU only.

## Materials and Methods

### Design

This study was part of the Professional Alliance with Clients in Treatment (PACT) study − a multi-site prospective naturalistic clinical cohort study among adolescents in outpatient YMHC and YAC. Two hundred and four consecutively admitted adolescents participated in the study using a − non-randomized − between-group A/B design. Treatment in group A consisted of TAU in 127 participants, and treatment in the subsequently admitted group B consisted of TAU plus a client feedback intervention (TAU + feedback) in 77 new participants. Assessments took place at the start of treatment and 2 and 4 months later. Youths in the TAU group A were admitted to treatment between April 2015 and September 2016. Following the last admission in group A, therapists were trained in how to conduct the feedback intervention between September and December 2016 (see details in the Methods section). Youths in the TAU + feedback group B were subsequently admitted between December 2016 and August 2018. The study was funded by The Netherlands Organization for Health Research and Development (No. 729101014) and approved by the Medical Ethical Board of the University Medical Center Leiden (P.15.001).

### Participants

**Fig. 1:**
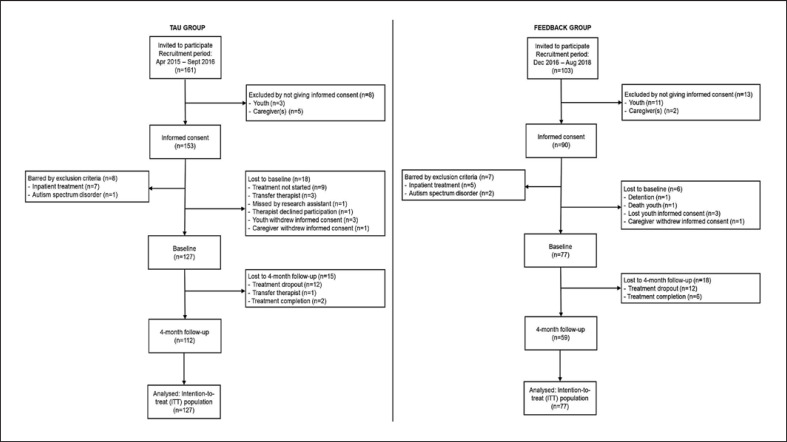
Flowchart per study group.

Eligible patients were 13–22 years old and started outpatient mental health or addiction treatment at one of the five participating treatment facilities in the Netherlands. Patients provided written informed consent for participation in this study. If under the age of 18 years, written informed consent was also obtained from the participants' parent/legal guardian/next of kin to participate in the study. Informed consent was provided by 243 youths (shown in Fig. 1). Patients were excluded from the study if they were cognitively incapable of comprehending the questionnaires (clinical judgment); if they needed inpatient treatment or a crisis intervention; or if they were diagnosed with a DSM-IV autism spectrum disorder (*n* = 15). The intent-to-treat (ITT) sample (TAU group: *n* = 127; TAU + feedback group: *n* = 77) consisted of all youths who completed the baseline assessment and started treatment. From these, 112 youths in the TAU group (88.2%) and 59 youths in the TAU + feedback group (76.6%) completed the 4-month follow-up assessment. Reasons for study termination are displayed in Figure 1. Twelve participants (9.4%) in the TAU group and twelve in the TAU + feedback group (15.6%) left treatment prematurely. Only one (8.3%) of the twelve non-completers in the TAU + feedback group had received the client feedback intervention in at least four treatment sessions. Two participants (1.6%) in the TAU group and 6 (7.8%) in the TAU + feedback group completed therapy before the 4-month follow-up. There were no significant differences in baseline mental health problems, substance use problems, and therapeutic alliance between treatment completers and non-completers.

### Interventions

#### Treatment as Usual

Patients in both study groups were offered TAU. For most patients, TAU consisted of an individual outpatient cognitive behavioral intervention, and the remaining patients received family-based treatment or some other treatment, e.g., psychomotor therapy and other psychotherapy (see Table 1).

#### Client Feedback Intervention

In the second study group B, patients received the client feedback intervention as an add-on to TAU for 4 months. Prior to the start of the TAU + feedback treatment, therapists received an intensive training provided by experienced staff in the client feedback intervention and motivational interviewing (MI).

Part of the training was dedicated to the client feedback intervention and the use of the ORS [14] and SRS [15, 22]. The ORS measures the domains of actual personal, interpersonal, social, and overall levels of the client's well-being, and the SRS measures client's feelings about the bond with his or her therapist, the topics that were discussed, the therapeutic tasks, and the perceived overall quality of the therapy session. Client feedback is needed to optimally match the treatment with the needs and preferences of the youth, although many clients find it difficult to express negative feedback about the therapy or the therapist and may choose to conceal their dissatisfaction [23, 24]. Therefore, besides introducing, scoring, and interpreting the ORS and SRS rating scales, the therapists were explicitly instructed how to encourage feedback from the youth and create a safe feedback atmosphere.

The remaining part of the training was dedicated to the use of MI techniques while eliciting and discussing the ORS and SRS ratings in the client feedback intervention. Before the training, all therapists had completed an MI e-learning course. MI is a cooperative and a goal-oriented conversation style with a focus on the client's motivation and commitment for behavioral change by triggering and exploring one's own reasons for change. Because MI pays great attention to ambivalence, friction, and dissonance in the therapeutic alliance in a collaborative and nonauthoritarian way, those conversation techniques are particularly suitable in eliciting and discussing client feedback.

At the end of the training, we distributed summaries of the course material and MI booster training materials. Finally, the trainers and research team were constantly available for consultation.

In providing the client feedback intervention, therapists introduced, administered, and discussed the (paper-pencil versions of the) ORS and SRS at the start and end of each treatment session, respectively. During each treatment session, therapists elicited their client's feedback according to principles derived from MI [25] and client-informed feedback manuals [26]. At the start of each session, the therapists directly interpreted the ORS scores (using a scoresheet and the clinical cutoff score of 28), discussed these scores with the youth, and used it as an agenda for that therapy session. In case of improved ratings on the ORS, therapists and youths discussed the context of progress. At the end of each session, the therapists directly interpreted (using a scoresheet and the clinical cutoff of 36) the SRS scores and discussed what should be maintained and what could be better or different in the subsequent sessions. Furthermore, therapists received, after every 3 sessions, a graph with plotted ORS and SRS scores from the research assistant. If the ORS scores showed no improvement or a decrease, therapists were trained to discuss the cause with their client and review the SRS scores to detect possible problems in the therapeutic alliance. In case therapists observed a persistent lack of progress, they were instructed to discuss the situation and inform their clients about possible strategies to improve results. Based on shared decision-making, therapists and youths could decide together to change the treatment intensity, strategy, or activities or to switch therapists. Because of the risk of social desirability in SRS scores, therapists were trained to create a safe feedback atmosphere and challenge the youth to express negative feedback to optimally match the youth's treatment needs.

Likewise, youths in the TAU-only group completed the ORS and SRS at each treatment session, but the ratings were not provided to the therapist. Instead, youths put their completed ORS-SRS form in a sealed envelope which was not accessible to the therapists. This way, the active component of client feedback, providing and discussing the ORS and SRS ratings, occurred only in the TAU + feedback group.

### Instruments and Assessments

At baseline, we collected data about participants' demographic background. At baseline and at 2 and 4 months follow-up, we assessed patients' mental health and substance use problems as well as patient- and therapist-rated therapeutic alliance with independent outcome measures that were not part of the client feedback intervention.

Mental health problems were assessed using the Strengths and Difficulties Questionnaire (SDQ) [27, 28], a commonly applied instrument to screen and monitor psychosocial problems in children and adolescents. The questionnaire contains 20 items focusing on difficulties that can be rated on a 3-point Likert scale ranging from “not true” (0) to “certainly true” (2). We used the SDQ total difficulties score (range 0–40), with higher scores indicating more problems. To assess the frequency of youths' primary substance use or behavioral addiction (gaming/gambling) in the past month, we used the substance use section of the Measurements in the Addictions for Triage and Evaluation, Youth version (MATE-Y) [29].

Therapeutic alliance was assessed with the 12-item version of the Working Alliance Inventory (WAI-12) [30, 31, 32]. Youths and therapists were required to rate each item on a 5-point Likert scale ranging from “never” (1) to “always” (5). We used the mean score on the WAI as an indication of the overall therapeutic alliance quality with higher scores indicating better quality of the alliance.

Furthermore, we registered the number of face-to-face sessions in both study groups. In the TAU + feedback group, we additionally registered the number of submitted and discussed ORS-SRS scales for each client and calculated the ratio of submitted and discussed ORS and SRS to the number of attended face-to-face sessions.

### Outcome Measures

We predefined a dichotomous outcome measure reflecting a favorable versus unfavorable treatment outcome status after 4 months as the primary outcome measure for youths in both YMHC and YAC. We opted for this short-term treatment outcome because most of the symptom improvement occurs in the first months of treatment [9, 33, 34]. For youth in YMHC, this treatment outcome status was based on the SDQ. Derived from procedures suggested by Jacobson and Truax [35] and De Beurs et al. [36], we defined an SDQ total score above the cutoff score of 12.5 as unfavorable. For youth in YAC, the treatment outcome status was based on the number of days of primary substance use or primary problem behavior − gaming or gambling − in the preceding 30 days. We followed the guidelines for routine outcome monitoring in Dutch addiction care [37] and defined 5 or more days of the primary substance use or gaming/gambling in the preceding 30 days, as an unfavorable treatment outcome status.

### Analyses

Given that treatment outcome scores and therapeutic alliance data were nested within three levels of clustering (i.e., study group, treatment facility, and therapist), we intended to use multi-level modeling. However, due to the limited sample size at the levels of treatment facility (TAU: five treatment facilities and 4–11 therapists per treatment facility; TAU + feedback: five treatment facilities and 4–9 therapists per treatment facility) and therapist (TAU: 56 therapists, with 1–8 patients per therapist; TAU + feedback: 31 therapists, with 1–8 patients per therapist), it was not possible to estimate effects accurately [38].

Therefore, in order to address our first study goal, pertaining to the effect of the client feedback intervention on treatment outcome, we conducted a logistic regression analysis with the treatment group (TAU vs. TAU + feedback) as the independent variable and our dichotomous treatment outcome status (favorable vs. unfavorable) as the dependent variable, controlling for baseline problem status and baseline between-group differences. Concerning the second study goal, pertaining to the effect of the intervention on youth-rated and therapist-rated alliance, we conducted regression analyses with the treatment group as the independent variable and including 4-month youth- as well as therapist-rated alliance as the dependent variable, with baseline alliance as the covariate.

Missing 4-month treatment outcome data (TAU: *n* = 15; TAU + feedback: *n* = 18) were estimated with a “best estimate” of the youth's outcome status provided by the treating therapist. Missing 4-month youth's and therapist's alliance outcome data (TAU: *n* = 15 and *n* = 20; TAU + feedback: *n* = 18 and *n* = 17, respectively) were imputed (20 sets). Imputations were calculated by including gender, age, treatment setting (YMHC or YAC), cultural background (Dutch or non-Dutch), baseline problem status (favorable or unfavorable) on the primary problem domain (mental health status for youth in YMHC; substance use status for youth in YAC), and, finally, baseline and 2-month youth- or therapist-rated alliance, respectively, as predictors.

The effects of the client feedback intervention were tested in the ITT population. Baseline characteristics between treatment groups were compared using χ^2^ tests for dichotomous variables and *t* tests or Mann-Whitney U tests for normally and non-normally distributed continuous variables, respectively. In sensitivity analyses, we estimated the effect of the client feedback intervention on 4-month treatment outcome status and therapeutic alliance in the per-protocol (PP) subpopulation, defined as participants who attended at least four treatment sessions (TAU and TAU + feedback groups), and − for participants in the TAU + feedback group − who had received the client feedback intervention in at least four treatment sessions.

Data processing, descriptive statistics, (logistic) regression analyses, and multiple imputations were conducted with SPSS, version 25.0. Exploration of multi-level modeling and calculation of pooled estimates for SDs from the multiple imputed datasets were performed in R version 3.5.1 using the packages “nlme” [39] and “MICE” [40].

## Results

### Patients

The baseline characteristics of the ITT population are summarized in Table 1. Overall, there were no significant differences between the two study groups in terms of age, gender, ethnicity, psychological complaints, and substance or behavioral addiction problems. From the primary non-substance use mental disorders among youths in mental health care, only eating disorder showed a significant difference between the two groups, with a higher rate of eating disorder in the TAU + feedback group than in the TAU group (χ^2^(1) = 8.2; *p* < 0.05). The TAU and TAU + feedback groups did not differ in first-session youth- and therapist-rated alliance.

### Therapist and Treatments

Fifty-six therapists participated in the TAU group (caseload range 1–8 patients; mean = 3.4), and 31 therapists were involved in the TAU + feedback group (caseload range 1–8 patients; mean = 3.65). Most therapists in the TAU + feedback group had also participated in the TAU group (24/31; 77.4%), and there were no significant differences in therapist characteristics between both groups (Table 1). The age of therapists ranged between 24 and 62 years (mean = 38.6 years; SD = 8.8). Most therapists were female (79.4%), with a Dutch cultural background (90.2%), a master's degree in social science (66.7%), and with work experience of 10 years or more (41.3%).

**Fig. 2:**
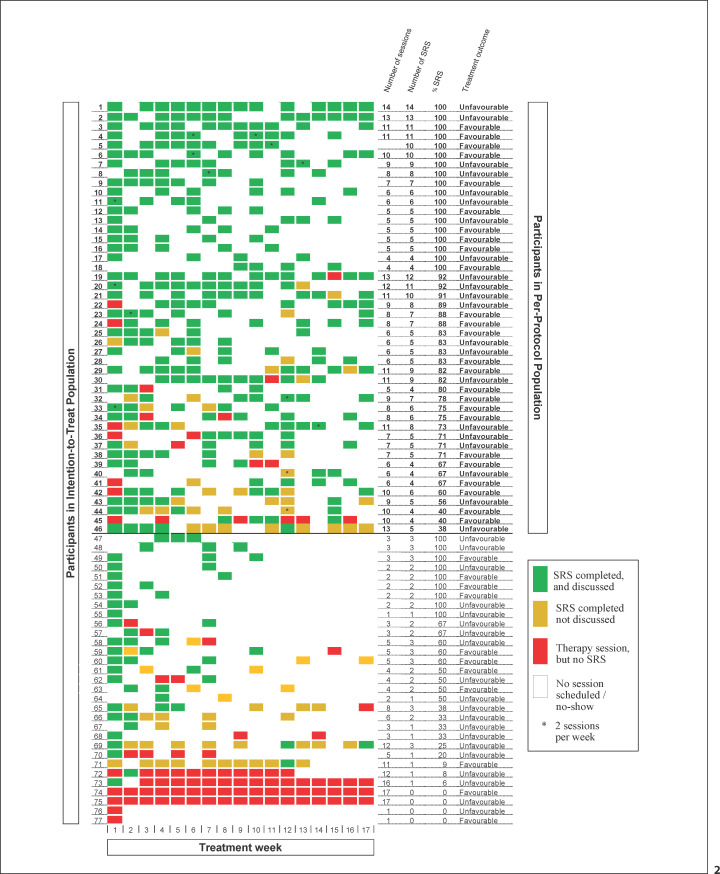
Treatment adherence, implementation fidelity, and treatment outcome at the participant level. Each row represents a participant, and each column (1–17) represents one treatment week. All rows represent the ITT population, and the rows above the bold line represent the PP subpopulation. The green rectangles represent a session with completed and discussed SRS; the orange rectangles represent a session with completed but not discussed SRS; the red rectangles represent a session with no SRS; the white rectangles represent no scheduled session or a no-show, and the * indicates two sessions in 1 week. Figure 2 shows that sixty-five youths attended at least four treatment sessions (65/77 = 84%; red, green, and orange rectangles) and 46 youths received the feedback intervention in at least 4 of these treatment sessions (46/77 = 60%; green rectangles).

Treatment duration and intensity were quite comparable between the two study groups; median treatment duration was 6 months (interquartile range [IQR] TAU: 4.5–8.0 months; IQR TAU + feedback: 3.8–8.0 months), and the median number of sessions attended was 7 (IQR: 4–11) in the TAU group and 6 (IQR: 4–10) in the TAU + feedback group. In the TAU group, 106 youths had attended at least four treatment sessions (106/127 = 83%). Youth treatment attendance and fidelity of the client feedback intervention in the TAU + feedback group are shown in Figure 2. Sixty-five youths attended at least four treatment sessions (65/77 = 84%; red, green, and orange rectangles), and 46 youths received the feedback intervention in at least 4 of these treatment sessions (46/77 = 60%; green rectangles). Therefore, the PP subpopulation consisted of 106 youths in TAU and 46 youths in TAU + feedback.

### Treatment Outcome Status

When youths in the TAU group and TAU + feedback group were combined, the proportion of youths with an unfavorable problem status decreased from 65.2% at baseline to 55.4% at 4-month follow-up (McNemar χ^2^ test, *p* = 0.01). As can be seen from the first row in Table 2, the proportion of youths in the ITT population with a favorable 4-month treatment outcome in the TAU + feedback group (46.8%) was slightly higher compared with the proportion of youths with a favorable 4-month treatment outcome in the TAU group (43.3%). A logistic regression analysis showed that this 3.5 percentage point difference in the favorable 4-month treatment outcome, controlling for baseline status and eating disorder, was not significant (*b* = 0.12; *p* = 0.71; OR 1.13; 95% CI: 0.60–2.12).

In the PP subpopulation, the proportion of youths with a favorable treatment status at month 4 was somewhat higher in the TAU + feedback group (54.3%) compared with the TAU group (49.1%), but the 5.2 percentage point difference, adjusted for baseline problem status and eating disorder, was not significant (*b* = 0.27; *p* = 0.53; OR = 1.31; 95% CI: 0.57–2.97).

In an additional post hoc analysis, we explored potential treatment effects when using the ORS outcome score as a dependent measure. This logistic regression analysis, adjusted for eating disorder, showed no significant difference in clinically significant improvement (i.e., ≥6 points improvement + ≥25 points) between the two groups (*b* = −0.52; *p* = 0.161; OR 0.59; 95% CI: 0.29–1.23).

### Therapeutic Alliance

The youth- and therapist-rated alliance scores for the ITT population are shown in the second and third rows of Table 2, respectively. Youth-rated alliance scores in the TAU group at month 4 (mean = 4.06, SD = 0.64) were comparable with youth-rated alliance scores in the TAU + feedback group (mean = 4.19, SD = 0.60). A linear regression analysis, using baseline youth-rated alliance and eating disorder as covariates, showed that youths in the TAU + feedback group did not report better alliance compared with youths in the TAU group (group [TAU vs. TAU + feedback]: beta = 0.10, *p* = 0.28).

Similarly, a linear regression analysis including baseline therapist-rated alliance and eating disorder as covariates indicated that therapist-rated alliance scores at 4-month follow-up in the TAU group (mean = 3.96, SD = 0.55) did not differ significantly from those in the TAU + feedback group (mean = 4.01, SD = 0.58) (group [TAU vs. TAU + feedback]: beta = −0.02, *p* = 0.808). Also, in the PP subpopulation, differences between the TAU and TAU + feedback groups in youth-rated alliance and therapist-rated alliance scores were not significant (beta = 0.13, *p* = 0.21, and beta = −0.01, *p* = 0.91, respectively).

## Discussion

In a sequential between-group design, we found that the addition of a client feedback intervention to TAU was not associated with improved alliance and treatment outcomes or less premature treatment drop-out among patients receiving youth mental health or substance use treatment. To our knowledge, this study is the first to investigate the effect of client feedback in YAC. Sensitivity analyses among the most adherent patients in both treatment groups did not show significant beneficial effects of client feedback nor did we find an effect on the ORS. These negative findings are contrary to our expectations because we showed in an earlier paper that strong initial − first-session − therapeutic alliance was robustly associated with a favorable treatment outcome 4 months later in our study group [11] and had expected that improving alliance by means of a systematic client feedback intervention would improve treatment outcomes correspondingly. Overall, however, our findings add to the largely negative findings reported in the Cochrane review of Bergman et al. [21] and call into question the potential efficacy of feedback interventions in youth mental health or addiction care.

Several factors may have accounted for our negative findings. As a first and foremost limitation, insufficient implementation of, and low adherence to, the feedback intervention may have caused, or contributed to, the absence of a significant treatment effect. Our study data show that less than two-thirds of the youths in the feedback group received four or more treatment sessions with feedback as intended, and even this threshold must be considered as quite lenient, given that a median of six sessions were attended in the feedback group. In most previous studies on the effects of client feedback in youth psychotherapy, details about implementation fidelity and treatment adherence were incomplete or not reported at all [21]. However, based on the available data, it appears that previous studies showed similar challenges regarding the implementation of the feedback interventions. In five of the six included studies in Bergman et al. [21], client feedback tools were used on a weekly basis and the intervention duration was relatively short, ranging between 1.8 session to 12 sessions. Only two studies [41, 42] provided further information about the implementation of the feedback intervention. In these studies, the number of sessions with completed feedback was higher than in our study, but the percentage of therapists that had received client feedback and thus could have used it in therapy was − like our study − quite low (approximately 30%).

Following the execution of our study, two studies on the effectiveness of formal client feedback in children and adolescents in Dutch mental health care were published. De Jong and colleagues [43] studied the effect of a feedback-informed treatment intervention and found a beneficial effect on quality of life − only for the group with 3–8 sessions and with no interaction with time − but no effect on symptom severity. No information was reported on the ratio of feedback-informed treatment sessions versus total number of sessions and to what extent therapists had discussed and used client feedback. In the second recent study, Van Sonsbeek and colleagues [44] examined the effects of three different types of feedback in 225 youths and found no significant differences between the different types of feedback on their primary (symptom severity) and secondary (quality of life) outcome measures. Notably, the authors argued that their negative findings may have been caused by implementation difficulties, unknown levels of receiving and sharing feedback, and clinicians' poor adherence to the feedback interventions. Taken together, these findings show that data about the implementation of and adherence to client feedback interventions are often lacking in publications and if reported suggest that successful implementation of feedback interventions is rarely achieved. We believe that our study findings are relevant even in the light of adherence problems as it adds to increasing evidence, indicating that systematic client feedback is challenging to implement effectively in typical outpatient mental health or addiction treatment facilities. Our detailed reported session-based visual representation of feedback frequency and treatment intensity may inform and encourage future studies on client feedback to report more details about implementation fidelity and treatment adherence.

Second, given that our study was not designed as a randomized controlled trial, initial − baseline − differences in patient characteristics or in TAU treatment offer between the first and the second youth sample could have accounted for lack of treatment effect. However, data show that patient characteristics at baseline did not differ between both study groups and neither did the underlying TAU offered in both treatment groups.

Lastly, our findings may reflect a “true” lack of treatment effect of the client feedback intervention. This may be the case but is at this point of time uncertain because implementation difficulties and poor adherence of patients as well as therapists in both our study and virtually all previous studies [21, 44] obscure a potential treatment effect.

## Conclusion

To conclude, despite intensive training, implementation, and monitoring, systematic client feedback added to usual treatment did not improve therapeutic alliance or treatment outcomes of youths in mental health and addiction treatment. Similar to virtually all studies of client feedback in youths, potential treatment efficacy in our study was obscured by poor adherence, low treatment fidelity, and high risk of bias. With Bergman et al. [21], we conclude that current evidence is insufficient to draw any conclusion about the benefits or lack thereof of client feedback in youth mental health or addiction treatment. Well-controlled studies with low risk of bias are highly recommended.

## Statement of Ethics

The study protocol was reviewed and approved by the Medical Ethical Board of the Leiden University Medical Center (P.15.001). Patients provided written informed consent for participation in this study. If under the age of 18 years, written informed consent was also obtained from the participants' parent/legal guardian/next of kin to participate in the study.

## Conflict of Interest Statement

The authors have no conflicts of interest to declare.

## Funding Sources

This research was funded by The Netherlands Organization for Health Research and Development (No. 729101014).

## Author Contributions

Renske Spijkerman and Vincent Hendriks designed the study with contribution from Albert Boon and Robert Vermeiren. Renske Spijkerman, Albert Boon, Robert Vermeiren, and Vincent Hendriks supervised the project. Patty van Benthem and Renske Spijkerman contributed to the implementation of the research. Patty van Benthem collected the data, performed the analysis, and drafted the manuscript. Peter Blanken designed Figure 2 and aided in interpreting the results. All authors discussed the results and commented on the manuscript.

## Data Availability Statement

The data that support the findings of this study are not publicly available because we did not request consent from study participants for using their coded (anonymous) study data for future research purposes. Further inquiries can be directed to the corresponding author.

## Figures and Tables

**Table 1:** Therapist and participant characteristics of the ITT population by the study group

	TAU group (*n* = 127)	TAU * feedback group (*n* = 77)
Demographic background		
Age (13–23), years, mean (SD), median	18.0 (2.5), 18.0	18.5 (2.6), 19.0
Male, %	51.2	46.8
Cultural background, non-Dutch, %	24.4	26.0
Education level low, %	62.2	66.2
Youth addiction care, %	44.1	53.3
Youth addiction care	*n* = 56	*n* = 41
Primary substance use disorder, %		
Cannabis use disorder	51.8	46.3
Alcohol use disorder	16.1	24.4
Gambling disorder	10.7	12.2
Hard drug use disorder	14.3	9.8
Gaming disorder	7.1	7.3
Days of primary substance use/problem behavior in the past month, mean (SD), median	14.3 (12.3), 13.0	12.5 (12.6), 8.0
Problematic substance use in the past month (≥5 days), %	62.5	56.1
Youth mental health care	*n* = 71	*n* = 36
Primary (non-substance use) disorder, %		
Anxiety disorder	26.8	30.6
Mood disorder	29.6	22.2
Eating disorder	−	11.1^a^*
Behavioral disorder	22.5	8.3
Attention-deficit hyperactivity disorder	7.0	5.6
Other disorders	14.1	22.3
SDQ		
SDQ score: 0–40, mean (SD), median	15.4 (5.4), 16.0	15.3 (4.8), 15.0
Problematic mental health status		
SDQ score ≥12.5, %	69.0	72.2
Treatment	*n* = 124	*n* = 77
Treatment type, %		
Cognitive behavioral interventions	75.0	75.3
Family-based treatment	5.6	2.6
Other	19.4	22.1
Concurrent pharmacological treatment: yes, %	17.9	15.6
Number of sessions, mean (SD), median	7.5 (3.9), 7.0	7.1 (3.9), 6.0
≥4 sessions, %	83.5	79.2
First-session therapeutic alliance		
Youth-rated WAI (1–5), mean (SD), median	3.9 (0.7), 4.1	4.0 (0.5), 4.0
Therapist-rated WAI (1–5), mean (SD), median	3.9 (0.5), 4.0	4.0 (0.5), 4.0
Therapist characteristics	*n* = 56	*n* = 31
Age (24–62), years, mean (SD), median	38.6 (9.4), 35.0	37.3 (8.7), 34.0
Male, %	29.9	23.4
Cultural background, non-Dutch, %	12.1	14.5
Master's degree in social science, %	68.9	81.3
Work experience of ≥10 years, %	42.5	39.0
Caseload (1–8)	3.4	3.7

SDQ, Strength and Difficulties Questionnaire.

a Using χ^2^ tests.

* *p* < 0.05.

**Table 2:** Treatment status and youth- and therapist-rated alliance for the TAU group and the TAU * feedback group in the ITT population and the PP subpopulation

	TAU group		TAU * feedback group	
	baseline (covariate)	4-month (outcome)	baseline (covariate)	4-month (outcome)
ITT population	(*n* = 127)	(*n* = 127)	(*n* = 77)	(*n* = 77)
Favorable treatment status	33.9%	43.3%^a^	36.4%	46.8%^a^
Youth-rated alliance, mean (SD)	3.92 (0.66)	4.06 (0.64)^b^	3.99 (0.49)	4.19 (0.60)^b^
Therapist-rated alliance, mean (SD)	3.91 (0.51)	3.96 (0.55)^b^	4.00 (0.53)	4.01 (0.58)^b^
PP population	(*n* = 106)	(*n* = 106)	(*n* = 46)	(*n* = 46)
Favorable treatment status	36.8%	49.1%^c^	39.1%	54.3%^c^
Youth-rated alliance, mean (SD)	3.94 (0.63)	4.05 (0.70)^d^	4.04 (0.51)	4.22 (0.71)^d^
Therapist-rated alliance, mean (SD)	3.93 (0.48)	3.98 (0.55)^d^	3.98 (0.51)	4.02 (0.55)^d^

Missing 4-month treatment status data were estimated with a “best estimate” of the youth's outcome status provided by the treating therapist. Missing 4-month youth's and therapist's alliance data were imputed.

a Estimated treatment status data − TAU: *n* = 15; TAU * feedback: *n* = 18.

b Imputed youth's and therapist's alliance data − TAU: *n* = 15 and *n* = 20; TAU * feedback: *n* = 18 and *n* = 17, respectively.

c Estimated treatment status data − TAU: *n* = 4; TAU * feedback: *n* = 5.

d Imputed youth's and therapist's alliance data − TAU: *n* = 4 and *n* = 9; TAU * feedback: *n* = 5 and *n* = 4, respectively.
